# The Cerebrospinal Fever Epidemic of 1917 at “X” Depot

**Published:** 1918

**Authors:** 


***The Cerebrospinal Fever Epidemic of 1917 at " X " Depot.*** By J. A. Glover, R.A.M.C. Abs. from the *Jour. R.A.M.C.*, January, 1918.

The author reviews the history of meningitis at his dépôt, emphasizing especially the relationship of the carrier rate[1] to the epidemic prevalence of the disease.

Type II meningococcus was responsible for most of the cases.

All cases occurred during exceptionally cold weather and in periods of undue crowding.

The rise in the carrier rate, as indicated by swabbings, was a premonitory sign of the outbreak of the disease. The danger rate, 20 per cent. (War Office Memorandum on Cerebrospinal Fever, p. 2) was reached six days before the first case occurred.

During the epidemic, the carrier rate among contacts and non-contacts was practically the same (about 34 per cent.).

1. See *Medical Bulletin* No. 5, March, 1918, Gordon's discussion of the carrier rate.

The use of the Levick spray for steam zinc sulphate solution, brought about a satisfactory drop in the carrier rate and a temporary cessation of cases in each instance.

The author points out, furthermore, that many of the patients (about 60 per cent.) were in their first month of service and that 40 per cent. had been inoculated or vaccinated within seven days of the onset (three on the same day).

Epidemics of German measles, influenza, and bronchitis appeared to help in the distribution of the disease, partly by coughing and sneezing and partly by reducing the vitality of the patients.

The author outlines the measures of prophylaxis adopted in 1918. In August the carrier rate fell to 2 per cent. thus affording the best opportunity to begin prophylaxis.

The measures adopted are :

1. Spacing beds (minimum interval 2 1/2 feet).
2. Special ventilation.
3. During the danger months (December, to March) antityphoid inoculation is done in the second month and vaccination during the first month.
4. A spray chamber has been established equipped with an external boiler and 20 Nines jets, thus obviating the disadvantage of the Levick spray.
5. Each regiment is sprayed in rotation every day for six days once a month, whenever there is a serious rise in the carrier rate. Recruits are sprayed for six days upon arrival.
6. A large sample swabbing (100 men) is taken weekly.

